# Intensive psychotherapy and case management for Karen refugees with major depression in primary care: a pragmatic randomized control trial

**DOI:** 10.1186/s12875-020-1090-9

**Published:** 2020-01-28

**Authors:** Andrea K. Northwood, Maria M. Vukovich, Alison Beckman, Jeffrey P. Walter, Novia Josiah, Leora Hudak, Kathleen O’Donnell Burrows, James P. Letts, Christine C. Danner

**Affiliations:** 1 Center for Victims of Torture, 2356 University Ave W Ste 430, St Paul, MN 55114 USA; 2 University of Minnesota, Boyton Health Mental Health Clinic, 410 Church St SE, Minneapolis, MN 55455 USA; 3 Live-Oak, 1300 W Belmont Ave #300, Chicago, IL 60657 USA; 4 HealthEast Roselawn Clinic, 1983 Sloan Pl #1, St Paul, MN 55117 USA; 5 St Joseph’s Family Medicine Residency Program, University of Minnesota Physicians, Bethesda Family Medicine Clinic, 580 Rice St, St Paul, MN 55103 USA

**Keywords:** Refugees, Depression, Primary care, PTSD, Basic needs, Case management

## Abstract

**Background:**

Despite an unparalleled global refugee crisis, there are almost no studies in primary care addressing real-world conditions and longer courses of treatment that are typical when resettled refugees present to their physician with critical psychosocial needs and complex symptoms. We studied the effects of a year of psychotherapy and case management in a primary care setting on common symptoms and functioning for Karen refugees (a newly arrived population in St Paul, Minnesota) with depression.

**Methods:**

A pragmatic parallel-group randomized control trial was conducted at two primary care clinics with large resettled Karen refugee patient populations, with simple random allocation to 1 year of either: (1) intensive psychotherapy and case management (IPCM), or (2) care-as-usual (CAU). Eligibility criteria included Major Depression diagnosis determined by structured diagnostic clinical interview, Karen refugee, ages 18–65. IPCM (*n* = 112) received a year of psychotherapy and case management coordinated onsite between the case manager, psychotherapist, and primary care providers; CAU (*n* = 102) received care-as-usual from their primary care clinic, including behavioral health referrals and/or brief onsite interventions. Blinded assessors collected outcomes of mean changes in depression and anxiety symptoms (measured by Hopkins Symptom Checklist-25), PTSD symptoms (Posttraumatic Diagnostic Scale), pain (internally developed 5-item Pain Scale), and social functioning (internally developed 37-item instrument standardized on refugees) at baseline, 3, 6 and 12 months. After propensity score matching, data were analyzed with the intention-to-treat principle using repeated measures ANOVA with partial eta-squared estimates of effect size.

**Results:**

Of 214 participants, 193 completed a baseline and follow up assessment (90.2%). IPCM patients showed significant improvements in depression, PTSD, anxiety, and pain symptoms and in social functioning at all time points, with magnitude of improvement increasing over time. CAU patients did not show significant improvements. The largest mean differences observed between groups were in depression (difference, 5.5, 95% CI, 3.9 to 7.1, *P* < .001) and basic needs/safety (difference, 5.4, 95% CI, 3.8 to 7.0, *P* < .001).

**Conclusions:**

Adult Karen refugees with depression benefited from intensive psychotherapy and case management coordinated and delivered under usual conditions in primary care. Intervention effects strengthened at each interval, suggesting robust recovery is possible.

**Trial registration:**

clinicaltrials.gov Identifier: NCT03788408. Registered 20 Dec 2018. Retrospectively registered.

## Background

The global refugee and migration crisis has reached unprecedented numbers, prompting the World Health Organization (WHO) to release a 2019–2023 Global Action Plan [1] that calls on health systems to adjust more rapidly to a new reality in which one out of seven persons worldwide is a migrant or refugee [2]. When refugees or migrants do receive health care, it is overwhelmingly in frontline settings such as humanitarian crisis arenas and primary care. Among other priorities, WHO’s plan emphasizes the urgent need to mainstream refugee and migrant healthcare services, promote a range of short-term and long-term interventions, address the social determinants of health, and integrate mental health within refugee healthcare provision.

Refugee populations exposed to war and torture have elevated rates of depression, PTSD, anxiety, chronic pain, and acute medical conditions [3–7]. Karen refugees from Burma have high rates of torture and war trauma related to a 70-year armed conflict with the government of Burma [8], and the health effects of these experiences have been compounded by lengthy stays in refugee camps on the Thai-Burma border without legal access to medical care, education, employment, or adequate food [9]. Newly resettled refugee populations present in primary care settings with a host of complex, interrelated biopsychosocial needs and profound access barriers involving culture, language, transportation, and health literacy [10–13]. These barriers, in concert with severe trauma and years of medical neglect pre-resettlement, create challenges for primary care clinics regarding elevated risk of severe disease, poor health outcomes, and high need for already limited clinic resources. Existing research [14–16] has described gaps in care for refugees navigating the U.S. medical system in particular.

While a strong body of evidence has supported the integration of behavioral health services into primary care to treat depression [17–21], anxiety [22, 23], and chronic health conditions [24, 25], no randomized control trials (RCTs) have investigated the efficacy of integrated services for refugee populations. Few trials exist in the field of refugee health due to the ethical and practical challenges of conducting experimental research with this population [26, 27]. Research has been constrained by small sample sizes, uneven comparison group sizes, singular outcomes, lack of control groups, lack of randomization, and non-blind assessment [3, 28].

RCTs are an essential component of establishing evidence of effectiveness. However, few RCTs resemble real-world clinical practice conditions or populations, which compromises both their applicability and credibility with practitioners [29–32]. For example, in a literature review of RCTs that examined representation of patients from “everyday clinical practice” in mental health, cardiology, and oncology, the authors conclude that a high proportion of the general disease population is excluded from trials, usually for reasons related to excluded patients’ higher risk profile or co-morbidities [30]. Additionally, in RCTs of behavioral health interventions, length of treatment is often far shorter and breadth of outcomes assessed is narrower than it is in real-world psychotherapy practice [32]. There are no known RCTs of intensive patient-centered behavioral health treatments for refugees in primary care lasting longer than 6 months that address the broad array of presenting psychological and social problems that resettled refugees face. Patient-centered is defined here to mean the patient chooses goals to work on, preferred means of achieving goals (among options offered and guided by their psychotherapists and case managers), and the pace of self-directed change. Behavioral health services for refugees must be flexible and robust enough to address each individual’s needs for psychosocial stabilization and each individual’s trajectory in overcoming the debilitating psychological consequences that commonly follow catastrophic losses and traumatic events. Experienced refugee mental health providers are guided by a repository of general principles, knowledge, and skills in cross-cultural trauma-informed practice [11, 26, 27, 33, 34] integrating various evidence-based components as opportunity and resources allow. There is dire need for pragmatic yet rigorous research on this type of real-world clinical model implemented in a primary care setting with refugee patients [35, 36].

We sought to examine the efficacy of behavioral health interventions located within the primary care setting that were congruent in length and flexibility to those provided in more specialist centres. We conducted a pragmatic RCT on a one-year behavioral health intervention consisting of psychotherapy and case management provided by refugee trauma specialists from the Center for Victims of Torture (CVT) within two urban primary care clinics serving Karen refugees from Burma, one of the largest refugee populations to arrive recently in our service area of Minnesota, USA [37].

The aim of the present study was to evaluate the benefits of intensive, coordinated psychotherapy and case management in primary care on common symptoms (depression, anxiety, PTSD, pain) and social functioning in refugees, relative to a comparison group who received care as usual from their primary care provider and usual referrals for mental health services.

## Methods

### Study design and oversight

A parallel-group randomized control trial was conducted with simple random allocation to either: 1) intensive psychotherapy and case management (IPCM) within the primary care clinic, or 2) care as usual (CAU), the clinic’s usual process for managing mental health concerns including referring to community providers or providing brief, onsite behavioral health support.

Ethical trial conduct and safety were overseen and approved by the institutional review boards of the University of Minnesota, Healtheast, and the Minnesota Department of Human Services.

### Pragmatic design

The growing call for more pragmatic studies [30] has led to increased analysis of their complexity. In any study, multiple components of design exist on continuums of highly pragmatic (i.e., Does the intervention work under usual conditions?) to highly explanatory (i.e., Does the intervention work under ideal, highly controlled conditions?) [38]. Accordingly, the key instrument framing conversations about pragmatic trials, the Pragmatic-Explanatory Continuum Indicator Summary (PRECIS-2) [39], uses a wheel format to capture a trial’s scores on nine domains that affect external validity, on one end of the continuum, and isolation of precise causal mechanisms, on the other. These domains include how representative the study is of real-world conditions in participant *eligibility criteria*, *recruitment* path, care *setting*, *organisation* (expertise or resources required), *flexibility in care delivery*, *flexibility in patient adherence*, intensity of *follow-up*, relevance of *primary outcomes* to patients, and inclusiveness of the *primary analyses* [39]. Refugees with high trauma exposure, insecurity of basic needs and high mobility, complex physical and mental health presentations, and multiple healthcare access barriers make a compelling case for studies to be highly pragmatic in order to be applicable to primary care settings charged with their care.

The present study’s design was pragmatic on seven of nine PRECIS-2 domains: (1) few *eligibility* criteria: adult Karen refugees from Burma, ages 18–65, with a diagnosis of Major Depressive Disorder (MDD); (2) *recruitment* occurred at the time of presentation and by primary care physician referral; (3) actual primary care *setting*; (4) providers had high *flexibility in delivering the intervention* of psychotherapy and case management according to patients’ self-chosen treatment goals; (5) patients had complete *flexibility in adherence to the intervention*; (6) the *primary outcomes* were highly relevant to refugee patients, representing common presenting symptoms and areas of social functioning CVT clinicians routinely address; and (7) data *analyses* included all available data using intention to treat. The two domains yielding a less pragmatic score were *organization* (we used highly skilled refugee behavioral health providers) and *follow-up* (although patients in both groups were followed with the same intensity of measurement, IPCM patients received a greater number of visits than is typical of behavioral health services in a U.S. primary care setting). Overall the study was thus pragmatic except in two areas integral to our understanding of effective treatment for this vulnerable population.

### Patients, setting, and location

**Fig. 1:**
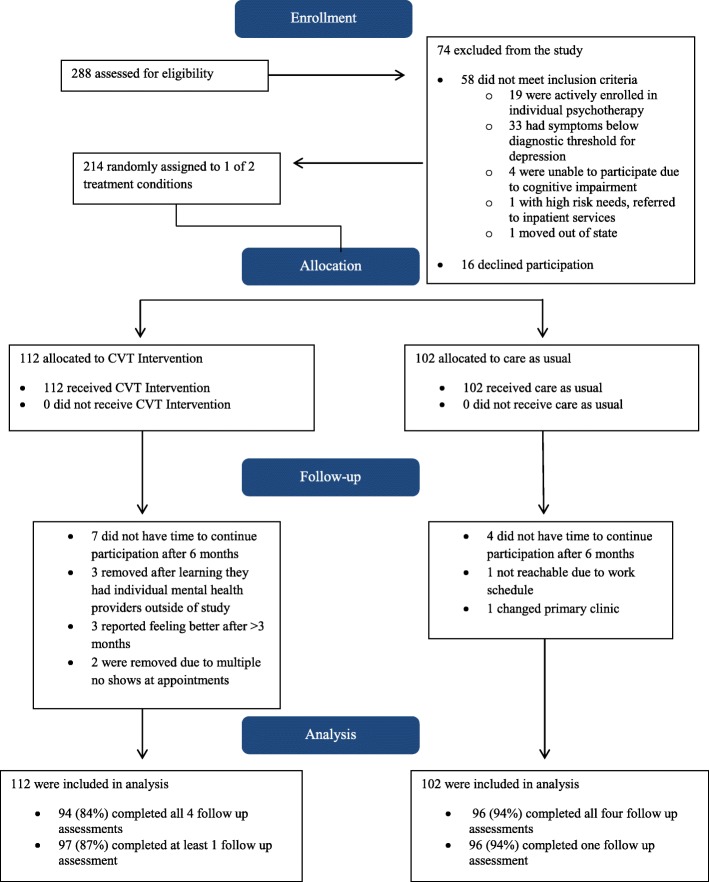
Consort flow chart of Karen refugee patients through the RCT. Patients referred to the study and enrolled in the intervention or care-as-usual groups from eligibility determination through baseline, 3, 6, and 12 months follow up

Recruitment occurred on a rolling basis from May 2013 – January 2017; data collection concluded in January 2018. Primary physicians at both clinics were aware of the parameters of the study as a randomized control trial for Karen refugee patients with depression and that eligibility (including current depression diagnosis) would be determined independently by a study clinician regardless of the patient’s current or past diagnoses. Karen refugee patients were referred by their primary physician based on the presence of depression symptoms (including unremitting pain as a common manifestation of depression in refugees [3–7]) in two urban primary care clinics in St Paul, Minnesota, USA. Potential participants were invited to meet with a study clinician and a professional interpreter who explained the study and obtained informed consent. For those participants who requested time to consider whether they wanted to enroll, the study clinician made arrangements to follow up with them at a later date, usually a week later. A Consort flow chart [40] of patients through the study is presented in Fig. 1.

After obtaining informed consent, the clinician conducted an eligibility assessment comprising the major depressive episode section of the Structured Clinical Interview for DSM-IV (SCID) [41], psychosis screening questions, and the CAGE-AID [42] for substance use. Inclusion criteria were: Karen refugee, ages 18–65, meets criteria for MDD according to SCID interview (MDD criteria did not change in DSM-V). Exclusion criteria were: current enrollment in individual psychotherapy or mental health case management [43], active psychosis that study providers determined was not culturally derived or trauma-related (many patients had psychotic-like symptoms such as seeing shadows and ghosts that were normative cultural expressions of distress and these were not excluded), chemical dependency or reported problems with non-prescribed drugs or alcohol on the CAGE-AID, and acute need at the time of screening for a higher level of care than the study provided (e.g., inpatient treatment). Ineligible patients received alternative referrals, and the referring physician was informed by message in the electronic health record so that care as usual could proceed in a timely manner.

### Randomized allocation

A coin toss by a research assistant otherwise uninvolved in the study was used to determine group allocation. Outcome assessors (research staff not involved in the intervention who administered the measures) were blind to group assignment.

### Intervention group

IPCM patients received services from both a psychotherapist and a case manager for 1 year. Depending on patient availability, appointments were weekly or bi-weekly and lasted 45 min – 1 h. A professional interpreter was utilized unless the provider was a native Karen speaker.

CVT psychotherapists providing the intervention included 3 doctoral-level psychologists and 1 master-level clinical social worker. CVT case managers included 4 master-level social workers and 1 bachelor-level social worker. Additional training for clinical staff included: completion of a 10-module blended learning course created by CVT called Fundamentals of Providing Services to Torture Survivors [44]; Adult Mental Health Targeted Case Management training provided by the state of Minnesota [45]; individual clinical supervision conducted by senior CVT psychotherapists; participation in monthly psychological consultation and clinical social work group supervision with other CVT providers, and up to 60 h of yearly continuing education training per individual provider.

Consistent with pragmatic randomized trial design to examine real-world practice with refugees [26, 29], providers delivering psychotherapy and case management tailored appropriate trauma and depression interventions to individual patients. Case management’s function was to help patients gain access to medical, social, educational, vocational and other necessary services connected to their mental health needs [46]. Case management interventions focused on re-establishing safety and stabilization [33, 34, 47]; facilitating communication, problem-solving and understanding between patients and medical providers [48]; and increasing skill in navigating health and community systems in resettlement [48]. Each patient and his/her case manager developed and worked from an Individual and Community Support Plan (ICSP) [46] that prioritized 3–5 goals, stated in the patient’s words (e.g., “I want to work to help my family with bills”; “I want to become U.S. citizen”). Core components of the case management are described in Table 1.

**Table 1:** Functions and Components of Psychotherapy and Case Management Intervention

Case Management	
Function: assist patients to gain access to medical, social, educational, vocational and other necessary services connected to their mental health needs Components:	
• Assessing patients’ needs and goals and impact of mental illness, and incorporating patients’ strengths and progress toward goals • Planning goals and goal-related steps, updating the individual and community support plan, finding new resources • Referring and linking to resources, supports and services • Coordinating with medical providers, community resources and natural supports identified by each patient as important to his or her recovery process • Monitoring the effectiveness of the resources, supports and services being utilized, especially with respect to refugees navigating health and community systems in resettlement • Discussing the progress made toward goals • Advocating as case managers on behalf of the patients’ mental health needs with medical, legal and social systems	
Psychotherapy	
Function: increase patients’ coping skills and understanding of their symptoms; alleviate symptoms and their impact Components:	
• Facilitating mind-body awareness; teaching and practicing relaxation skills • Providing psychoeducation on the relationship between trauma/stress and symptoms, treatment options for mental health symptoms, use of medications, and the doctor-patient relationship in Western medical culture • Developing and teaching compensatory strategies for taking medications accurately and following health plan instructions that accommodate impairments in memory/concentration and other mental health symptoms • Applying evidence-based trauma-focused treatments to reduce symptoms of depression, anxiety, and posttraumatic stress • Problem-solving with patients to decrease impact of symptoms and distress by changing coping behaviors and thought patterns • Advocating as psychotherapists on behalf of patients’ mental health needs with medical, legal and social systems	

Psychotherapy functioned to increase patients’ coping skills and understanding of their symptoms, as well as to alleviate these symptoms and their impact. Psychotherapists taught mind-body awareness and relaxation skills calibrated to survivors of severe trauma and catastrophic losses. They provided education about the connections between trauma/stress and symptoms, use of medications and normative expectations for the doctor-patient relationship in Western culture (e.g., medications are not shared; dosage is not changed safely without consultation with one’s doctor; patient is expected to raise concerns proactively rather than waiting to be asked, etc.), and compensatory strategies for patients with significant memory or concentration impairments to take their medications accurately and follow behavioral aspects of their health care plan. Psychotherapists applied evidence-based treatments for PTSD and depression tested on refugee populations, including Narrative Exposure Therapy and Cognitive Behavior Therapy [3, 28]; they also utilised components of other psychoeducational approaches and trauma-focused treatments, such as Sensorimotor Psychotherapy [49], and patient-centered methods such as Motivational Interviewing [50]. Where psychological assessment and diagnoses had implications for needed services or benefits, psychotherapists advocated within medical, legal, and social service systems on behalf of individual patient needs related to mental health symptoms (e.g., completing waiver forms for the U.S. civics exam and English language requirement for U.S. citizenship, etc.). Common components of the psychotherapy are summarized in Table 1.

At the team level, CVT’s approach emphasized active interdisciplinary coordination and a relational focus anchored in cultural humility [51] to address survivors’ priorities as the primary architects of their healing and work together to co-construct meaning and behavioral change. CVT providers communicated frequently with one another and with patients’ primary care providers to address overarching themes and challenges in a patient’s care. CVT providers scanned their assessments and case notes into patients’ Electronic Medical Records (EMRs). CVT clinicians also communicated with physicians via the EMR and reviewed their patients’ EMRs with read-only access. When possible, clinicians communicated in-person with physicians spontaneously between patient sessions, in warm handoffs with patients, and in planned case consult meetings with physicians.

Trauma and loss were understood to have ongoing community-based sociopolitical and historical dimensions rather than being conceptualized as discrete past events that happened to individuals. Treatment was responsive to the instability and ongoing acculturation stressors in the lives of refugee patients requiring responses to multiple unplanned interruptions, including financial, housing, employment, family, and health-related crises. A Karen coordinator provided repeated, active follow-up to remind clients of appointments and overcome transportation and language barriers. Interventions addressed symptoms recognized by conventional biomedical culture and Karen idioms of distress identified by patients to their CVT providers. Common approaches used with the intervention group are described in a published toolkit for serving refugees in primary care settings [52].

### Control group

Participants in the control group received care as usual, without CVT involvement beyond administration of outcome measures. Once randomized, CAU patients could be referred to a full range of behavioral health services by their primary care physician. Use of behavioral health services by patients in the CAU groups was monitored by primary care providers but not by the study.

### Data collection and measurement

Demographic characteristics were collected prior to randomization. Pre-specified outcomes were mean change in depression, anxiety, PTSD, pain and social functioning scores over the year of enrollment. Outcomes were collected at baseline, 3, 6 and 12 months using instruments found to be reliable and valid with refugee populations. Presence and severity scores of symptoms associated with MDD and Generalized Anxiety were measured on a 4-point Likert scale using the Hopkins Symptom Checklist-25 (HSCL-25) [53]. Presence and severity of symptoms associated with PTSD were similarly measured using Part 3 (17 PTSD symptoms) of the Posttraumatic Diagnostic Scale (PDS) [54] adapted to assess DSM-V diagnostic criteria. Presence and severity of pain was measured using an internally developed 5-item Pain Scale with adequate internal consistency of α = .76. Social functioning in meeting basic needs, stabilization, employment, social support, adjustment, and community engagement was measured with a 37-item standardized instrument on a 7-point Likert scale validated with refugees [55]. Instruments were selected based on extensive research indicating high prevalence of depression, anxiety, PTSD and pain in refugee populations [3, 56, 57]. Torture, war, and resettlement also impact social functioning, including basic needs, legal status, social support and involvement, employment and education, and engagement with one’s geographic community. Measures were administered by a trained assessor, blinded to treatment condition, who followed scripted protocols and used a professional interpreter. Assessors had no contact with CVT providers to minimize breaches to blindness and bias. The only exception occurred when a participant expressed intent to harm self or others. In these instances, the protocol allowed for appropriate crisis response without breaching assessor blindness.

### Sample size

Power analysis was originally conducted a priori using depression symptom scores as the outcome variable based on assumption of a 20% attrition rate [58]. Due to lower than expected attrition (10%), we re-calculated a sample size of at least 95 in each treatment group (190 participants total) to detect statistical significance at the alpha <.05 level with power of 80% or greater.

### Analysis

Mean (SD) baseline characteristics of participants randomized to the intervention or control groups were analyzed using t-tests for continuous and chi square tests for categorical data (see Table 2). Standardized t-scores were created for all outcomes using the normed population distribution collected at CVT [59]. All dependent variables met the statistical assumptions of normality, independence, homoscedasticity and sphericity prior to inferential analysis. Treatment effects were examined through repeated measures analysis of variance. Comparisons between groups were pre-specified and all tests were two-sided. Pairwise comparisons were performed post hoc with Sidak adjustment for comparison of mean scores at each time point between groups. All analyses were conducted according to intention-to-treat methods [60]. An alpha cutoff of *p* ≤ .05 was used to assess statistical significance. Effect sizes were calculated using partial eta squared and interpreted as 0.010–.059 = small, 0.060–.139 = medium, > 0.14 = large [61]. Statistical analyses were conducted in Statistical Package for the Social Sciences (SPSS) 24 [62] and R version 3.4.4 [63].. As cases were allocated in a non-random way, weighing pragmatic concerns of case load, provider availability, and where possible gender matching, no analysis of provider-related differences was included in the analysis.

**Table 2:** Baseline Characteristics of Study Participants (*N* = 214)

	IPCM	CAU	Total Sample	*P*
Total- no. (%)	112 (52.3)	102 (47.7)	214 (100)	
Women- no. (%)	92 (82.1)	79 (77.5)	171 (79.9)	
Age^a^	43.84 + 3.13	41.77 + 3.91	42.76 + 3.28	.247
Unemployed-no. (%)	94 (83.9)	88 (86.3)	182 (85.0)	.607
Reported Torture-no. (%)	41 (36.6)	36 (35.3)	77 (35.9)	.311
Reported Direct Harm-no. (%)	78 (69.6)	66 (64.7)	144 (67.3)	.194
Education Completed^a^	2.9 + 3.12	2.6 + 3.67	2.8 + 3.39	.519
Number in Household^a^	6.01 + 1.74	6.24 + 2.02	6.13 + 1.92	.372
Length of Resettlement^a^	4.11 + 2.5^b^	4.6 + 2.10^b^	4.29 + 2.34	.124

^a^Plus-minus values are means + standard deviations

^b^Residual imbalances between groups in length of resettlement found in the raw data were adjusted for using propensity score matching with weighted regression

## Results

Of the 288 patients screened for eligibility, 58 did not meet inclusion criteria and 16 eligible patients declined to participate. Of the 58 ineligible patients, 33 did not meet criteria for MDD; 19 were already receiving individual psychotherapy or case management; 4 were unable to participate in psychotherapy due to cognitive impairment; 1 required inpatient psychiatric care not available through the intervention; and 1 patient moved to another state.

Overall, 214 participants were enrolled in the study and completed a baseline assessment. Of these 187 (87.4%) completed all four assessments; 193 (90.2%) completed the baseline and at least one follow up assessment.

Participant characteristics at baseline by treatment group are provided in Table 2, including gender, age, employment status, completed years of education, reported experiences of torture and harm resulting from war trauma, household size and length of time spent resettled in the United States. Propensity score matching was performed using weighted regression to adjust for residual imbalances in length of resettlement between treatment groups [64]. No statistically significant differences were identified between groups in length of resettlement post-matching. All other demographic differences between treatment groups measured at baseline were non-significant. Therefore, no additional adjustments were made for potential confounding variables in the repeated measures analysis of variance.

On average, IPCM participants received 41.27 + 16.70 psychotherapy sessions and 38.31 + 15.29 case management sessions during their 1 year enrollment in the study. Average symptoms at baseline among all participants met clinical cutoffs (average raw item score > 1.75) for depression and anxiety on the HSCL-25 [65] and were similarly elevated on the PDS. Outcomes in symptoms and social functioning over time are reported in Table 3.

**Table 3:** Changes in symptoms and functioning over time between IPCM (*N* = 112) and CAU (*N* = 102)

	CAU t-score Means + SD	IPCM t-score Means + SD	Between-group Difference (95% CI)	Partial eta squared^a^
Depression				
Baseline	52.78 + 5.25	52.66 + 5.53	.12 [− 1.34 to 1.57] NS	
3 months	52.03 + 5.79	47.90 + 6.07*	4.13 [2.53 to 5.73] ***	
6 months	51.24 + 6.74	46.87 + 6.24***	4.37 [2.62 to 6.12] ***	
12 months	51.42 + 5.51	45.96 + 6.27***	5.47 [3.87 to 7.06] ***	0.214
Anxiety				
Baseline	52.29 + 3.65	52.27 + 4.99	.02 [− 1.21 to 1.17] NS	
3 months	52.12 + 6.49	48.94 + 2.90***	3.18 [1.85 to 4.51] ***	
6 months	51.14 + 3.78	47.98 + 4.19**	3.16 [2.08 to 4.24] ***	
12 months	51.58 + 3.89	46.91 + 2.85***	4.67 [3.76 to 5.58] ***	0.193
PTSD				
Baseline	52.53 + 6.45	52.90 + 6.13	.37 [− 1.33 to 2.07] NS	
3 months	51.72 + 5.97	48.80 + 6.02***	2.92 [− 1.30 to 4.54] **	
6 months	50.75 + 5.46	47.45 + 6.61***	3.30 [1.66 to 4.94] ***	
12 months	51.39 + 5.91	46.76 + 6.52***	4.63 [2.95 to 6.31] ***	0.224
Pain				
Baseline	52.05 + 9.67	52.34 + 9.73	.29 [− 2.33 to 2.91] NS	
3 months	51.85 + 9.32	49.90 + 9.81*	1.9 [.63 to 4.53] NS	
6 months	51.70 + 10.26	49.68 + 9.45*	2.02 [.64 to 4.68] NS	
12 months	52.35 + 9.47	48.56 + 10.51*	3.79 [1.08 to 6.50] **	0.172
Basic Needs/Safety				
Baseline	47.75 + 5.24	48.06 + 5.52	.31 [− 1.14 to 1.76] NS	
3 months	47.51 + 5.79	51.54 + 6.06**	4.03 [2.43 to 5.63] ***	
6 months	48.67 + 6.74	51.72 + 6.25***	3.06 [1.30 to 4.82] ***	
12 months	49.43 + 5.51	52.63 + 6.28***	5.44 [3.86 to 7.05] ***	0.247
Immigration Stability				
Baseline	47.04 + 3.65	47.42 + 4.57	.04 [−.73 to 1.49] NS	
3 months	46.78 + 6.49	48.90 + 2.95*	2.21 [.74 to 3.50] ***	
6 months	48.12 + 3.78	50.36 + 4.21*	2.24 [1.16 to 3.32] ***	
12 months	48.34 + 3.89	50.67 + 2.91*	2.33 [1.40 to 3.26] ***	0.125
Employment				
Baseline	47.58 + 9.67	48.12 + 9.74	.54 [−2.08 to 3.16] NS	
3 months	47.71 + 9.33	50.69 + 9.81*	2.98 [.40 to 5.56] ***	
6 months	48.39 + 10.24	51.83 + 9.49*	3.44 [.77 to 6.11] ***	
12 months	47.80 + 10.91	52.36 + 10.45**	4.56 [1.68 to 7.44] ***	0.131
Social Support				
Baseline	47.86 + 6.41	47.05 + 6.17	.81 [−.88 to 2.51] NS	
3 months	49.51 + 5.97	51.48 + 6.03*	2.37 [.75 to 3.99]**	
6 months	49.34 + 5.48	51.39 + 6.61*	2.02 [.37 to 3.67]*	
12 months	49.91 + 5.79	51.84 + 6.53***	1.9 [.26 to 3.60]*	0.133
Cultural Adjustment				
Baseline	48.70 + 5.09	48.57 + 5.23	.13 [−1.26 to 1.52] NS	
3 months	47.56 + 5.04	51.29 + 5.31*	3.73 [2.33 to 5.13]***	
6 months	49.47 + 5.52	53.38 + 6.10**	3.91 [2.34 to 5.48]***	
12 months	50.19 + 5.98	54.27 + 6.02***	4.01 [2.38 to 5.62]***	0.14
Community Engagement				
Baseline	48.52 + 8.27	48.59 + 7.76	.07 [− 2.23 to 2.09] NS	
3 months	48.94 + 8.02	49.79 + 7.82*	.85 [− 1.29 to 2.99] NS	
6 months	49.91 + 7.89	52.75 + 7.71**	2.84 [.73 to 4.94]**	
12 months	50.13 + 8.26	53.10 + 8.05***	2.97 [.77 to 5.17]**	0.138

Abbreviation: *NS* Non significant

* *P* < .05

** *P* < .01

*** *P* < .001

^a^Effect size, reported as partial eta squared values, indicates the size of the differences observed between groups. 0.01 or more are small effects, 0.06 or more are medium effects, and 0.14 or more are large effects

### Intervention response

Statistically significant changes in symptoms were found between groups, and the mean differences between groups were large (see Table 3). IPCM participants demonstrated statistically significant mean reductions in depression, anxiety, PTSD, and pain symptoms from baseline to 3 months. Positive treatment effects continued through 12 months in all symptom outcomes for the IPCM group. In contrast, CAU participants demonstrated non-significant reductions in symptom outcomes over time. Mean differences between groups were statistically significant in 3, 6 and 12 month outcomes of depression, anxiety and PTSD. Mean differences between groups for 3 and 6 month outcomes of pain were non-significant; however, statistically significant differences were observed between groups for 12 month outcomes of pain.

Statistically significant changes in social functioning outcomes were observed between groups, and the mean differences were large for basic needs/safety and cultural adjustment outcomes. Mean differences between groups for immigration stability, employment, social support and community engagement outcomes were moderate (see Table 3). Statistically significant mean differences between groups were observed from baseline to 12 months in basic needs/safety, immigration stability, social support, cultural adjustment, and community engagement outcomes. Statistically significant mean improvements in basic needs/safety, social support, cultural adjustment and community engagement outcomes were observed for the IPCM group at each follow-up assessment. Non-significant mean differences were observed for immigration stability and employment outcomes between 6 to 12 months for the IPCM group. The CAU group demonstrated nonsignificant mean differences in all social functioning outcomes over time.

**Fig. 2:**
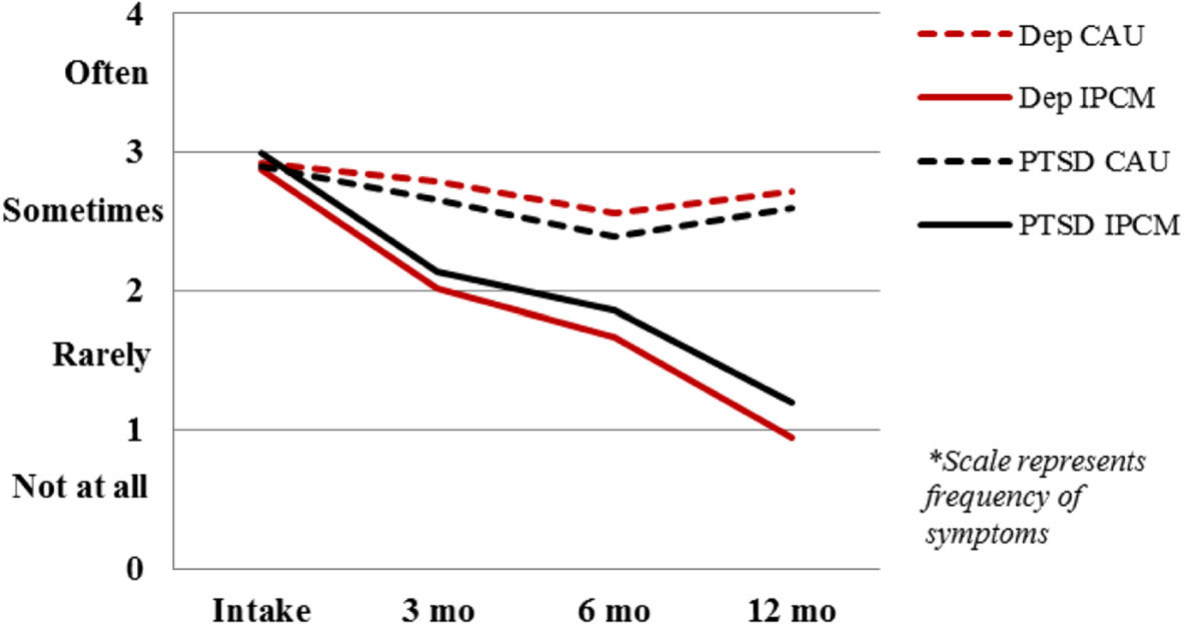
Change in depression and PTSD over time. Mean scores of patient symptom frequency using Hopkins Symptom Checklist-25 for depression (15 items) and Posttraumatic Diagnostic Scale PTSD symptoms (17 items) for Intensive Psychotherapy & Case Management and Care-As-Usual groups at baseline, 3, 6, and 12 months

High rates of depression and PTSD are well documented among refugees. Incremental changes in the frequency of depression and PTSD symptoms over the length of the trial are depicted for each group in Fig. 2.

**Fig. 3:**
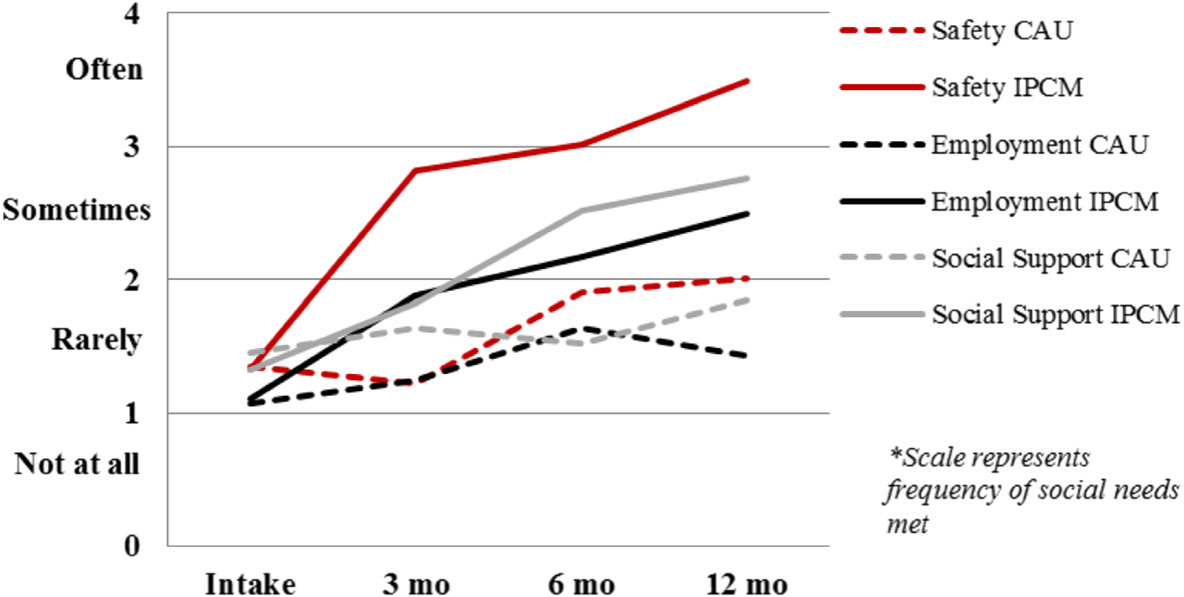
Change in meeting social needs over time. Mean scores of how often social needs are met on CVT Social Circumstances and Functioning Inventory subscales of Safety, Employment, and Social Support for Intensive Psychotherapy & Case Management and Care-As-Usual groups at baseline, 3, 6, and 12 months

Safety/basic needs, employment and social support are critical aspects of resettlement. Incremental changes in the frequency of met social needs in safety, employment and social support over the length of the trial are illustrated in Fig. 3.

## Discussion

In this pragmatic randomized control trial of 214 resettled adult Karen refugees with Major Depression receiving 1 year of psychotherapy and case management in a primary care clinic setting, patients demonstrated significant decreases in symptoms and significant increases in social functioning. These improvements were significant at 3 months, and additional positive treatment effects continued for the intervention group through the end of the intervention at 12 months, increasing in magnitude over time. Patients receiving care as usual, which potentially included behavioral health services provided onsite or in the community, did not significantly improve in reported symptoms or social functioning over 3, 6, and 12 months. The mean differences (effect sizes) observed between the intervention and care as usual groups were large for symptoms (depression, anxiety, PTSD, pain), meeting basic needs, and cultural adjustment; corresponding effect sizes were moderate for immigration stability, employment, social support, and community engagement.

To our knowledge, this study is the first of its kind in several respects. There have been no prior RCTs investigating the efficacy of behavioral healthcare integrated within primary care settings for refugees [35], much less RCTs that employ a pragmatic design to test an intervention that is congruent in length and flexibility with the care provided to refugees at specialist centres. These findings are important to the many specialized treatment centres operating in resettlement contexts including the United States, some of which offer services within hospital or primary care settings [66]. Prior research on behavioral health in refugees has focused on descriptive reports of symptomatology in response to pre- and post-migration stress and trauma [36]. Assessment of social functioning is rare, despite evidence that many protective factors for refugee health are social in nature [47, 67]. Clinical trials in refugee health are often limited by methodological weaknesses including small samples, non-random group assignment, non-blind assessment, and cross-sectional design; the most rigorous studies have evaluated a brief manualized treatment that would often be one component (e.g., cognitive processing therapy; narrative exposure therapy) of a more lengthy multidisciplinary treatment under real-world conditions (for recent examples, see [68–70]). This study has demonstrated that rigorous, pragmatic trials of behavioral health intervention can be effectively implemented in primary care to address the symptoms and functioning of refugee patients.

Primary care for refugees who have experienced trauma and catastrophic loss has presented unique challenges to physicians, particularly related to patient engagement, trust, and the treatment of chronic pain [11]. In this study, the delivery of intensive behavioral health services in the primary care clinic resulted in significant, sustained improvement across multiple areas of well-being. Our findings suggest that patients demonstrate greater improvements with more intensive psychotherapy and case management than is typically afforded in a primary care setting. Traditionally, integrated behavioral health services in primary care focus on brief assessment, brief treatment, and referral to other community based mental health services. This sample reflected common characteristics of refugee populations seeking medical care, as described in the literature: patients at intake reported high levels of torture and war trauma, post-trauma symptoms including pain, complex medical conditions, and unemployment. At baseline, patients reported substantial basic needs (food, shelter, housing) and low social support and cultural adjustment. And yet, the steady and clinically meaningful improvement demonstrated by patients receiving the intensive intervention for 1 year showed that remarkable progress is possible with sufficient resources in place.

### Limitations

Inherent in the design of a pragmatic RCT is the limitation with respect to isolating specific explanatory mechanisms [29]. This study does not examine which components of psychotherapy and case management were more strongly associated with improvements in symptoms and functioning. The amount, type and quality of non-CVT behavioral health interventions received by the care as usual group were not controlled for in the design.

While assessors were blind to study condition, primary care physicians and nurses could not be blinded as such, as coordination with a patient’s primary care team was inherent to the intervention studied. It is possible that this knowledge of patients’ conditions affected care in unknown ways, either biased for or against the intervention or care as usual.

Across conditions, most participants in this study were on multiple medications, including medications for depression, anxiety, sleep, and pain. This study was not resourced to measure prescription medications as taken, or not taken, or mis-taken, by refugees. Possible interactive effects of prescribed medications with this intervention, as well as traditional remedies used by refugee patients, will need to be explored in future research.

Given the paucity of controlled research with refugees, we chose in this study to focus on outcomes of symptom levels and adaptive functioning relevant to refugees that could be measured reliably by an assessor blinded to study condition. A limitation of the study is that we did not systematically measure other outcomes more tailored to the intervention group, such as types of goals developed under a patient-centered approach and the proportion that were met for the IPCM group. Results of a semi-structured interview that we administered to a subset of the IPCM group (*n* = 40) on active ingredients of the treatment from the patient’s perspective are published elsewhere [48].

Regular comprehensive assessments by compassionate, skilled assessors may have influenced care and the study cannot explain why the care as usual group did not significantly improve in symptoms or functioning; it was not designed to systematically examine patterns of difference within the CAU group. In a busy real-world setting, some clients may have reported receiving mental health services when in fact they were merely completing regular measures. Physicians would have had to check the patient’s electronic health record to verify this information and so may have been less likely to provide alternative referrals to a few CAU patients who wrongly reported receiving mental health services. Future studies parsing out what constitutes care as usual in refugee patients would do well to ensure via multiple channels that busy physicians are informed of study condition, tracking it, and not relying on patient report in a patient population with limited understanding of complex host-country services.

Other limitations of the study include gender imbalance and ethnic homogeneity. Eighty percent of participants were women. Gender differences and/or bias in reporting, assessing or treating mental health problems are discussed elsewhere [71] and may have been operative in this study. Although women and children are disproportionately represented in refugee populations [72], the findings may have less generalizability to men. For feasibility reasons, the study focused on one recently resettled refugee group: the Karen from Burma. Research with additional ethnic groups is needed to examine the efficacy of the intervention more broadly.

Finally, the benefits of not restricting treatment to a brief manualized protocol present corresponding challenges for replicability. We believe the value of examining principled, patient-centered services with delineatated functions and components by skilled refugee behavioral health providers exceeds the costs of not examining these non-manualized services and not making them more accessible to others through an evolving evidence base. Beyond the scope of this study, future research could examine a host of potential factors that might influence patient wellbeing or responsiveness to this type of intervention, including co-morbidities, prescribed medications, life events, other patient variables, and characteristics of the provider or intervention. Future pragmatic studies are needed to investigate the efficacy of this type of intensive intervention for a wider range of refugee patients in different care settings.

The implications for practice based on this study are that, despite the multidimensional complexities and challenges involved in their care, refugees can be well served by coordinated, intensive behavioral health interventions offered within the primary care setting. Primary care clinics serving large numbers of refugees can see better outcomes in these patients by offering intensive psychotherapy and case management services in the place of brief integrated behavioral health services or referral to community mental health.

## Conclusions

The study conducted the first known randomized trial on the effectiveness of integrated behavioral health in primary care for refugees with Major Depression. Karen refugees receiving psychotherapy and case management over a 1 year period demonstrated mental health symptom reduction, pain reduction, and improvements in social functioning. Effects of the intervention were observed to strengthen at each measured interval, suggesting cumulative gains.

## Data Availability

The datasets collected and analyzed during the current study are not publicly available to maintain the privacy and confidentiality of participants enrolled in the trial and per health care system regulations of the trial sites. Data are available from the corresponding author on reasonable request and permission of the Center for Victims of Torture, University of Minnesota Physicians Inc. and Healtheast Care systems.

## Abbreviations

CAU: Care as usual
CI: Confidence interval
CVT: Center for Victims of Torture
DSM: Diagnostic & Statistical Manual of Mental Disorders
HSCL-25: Hopkins Symptom Checklist-25
ICSP: Individual and community support plan
IPCM: Intensive psychotherapy and case management
IRB: Institutional review board
MDD: Major Depressive Disorder
PDS: Posttraumatic Diagnostic Scale
PRECIS-2: Pragmatic-Explanatory Continuum Indicator Summary-2
PTSD: Posttraumatic Stress Disorder
RCT: Randomized control trial
SCID: Structured Clinical Interview for DSM-IV
SD: Standard deviation
SPSS: Statistical Package for the Social Sciences
WHO: World Health Organization
